# Temporal Patterns of COVID-19-Associated Pulmonary Pathology: An Autopsy Study

**DOI:** 10.7759/cureus.20522

**Published:** 2021-12-19

**Authors:** George S Stoyanov, Nevena Yanulova, Lyuben Stoev, Nedyalka Zgurova, Viktoriya Mihaylova, Deyan L Dzhenkov, Martina Stoeva, Nadezhda Stefanova, Kalin Kalchev, Lilyana Petkova

**Affiliations:** 1 General and Clinical Pathology/Forensic Medicine and Deontology, Medical University of Varna, Varna, BGR; 2 Anatomy and Cell Biology, Medical University of Varna, Varna, BGR

**Keywords:** endotheliitis, squamous cell metaplasia, fibrosis, pulmonary morphology, lung changes, histopathology, autopsy, pathology, covid-19, sars-cov-2

## Abstract

Introduction

The novel coronavirus variant - severe acute respiratory distress syndrome coronavirus 2 (SARS-CoV-2) and the disease it causes clinically (novel coronavirus disease 2019 or COVID-19) have placed medical science into a frenzy due to the significant morbidity and mortality, as well as the myriad of clinical complications developing as a direct result of infection. The most notable and one of the most severe changes in COVID-19 develops in the lungs.

Materials and methods

All cases of real-time polymerase chain reaction (rtPCR)-proved COVID-19 subjected to autopsy were withdrawn from the central histopathology archive of a single tertiary medical institution - St. Marina University Hospital - Varna, Varna, Bulgaria. Pulmonary gross and histopathology changes observed on light microscopy with hematoxylin and eosin as well with other histochemical and immunohistochemical stains were compared with the time from patient-reported symptom onset to expiration, to compare the extent and type of changes based on disease duration.

Results

A total of 27 autopsy cases fit the established criteria. All cases clinically manifested with severe COVID-19. From the selected 27 cases, n=14 were male and n=13 were female. The mean age in the cohort was 67.44 years (range 18-91 years), with the mean age for males being 68.29 (range 38-80 years) and the mean age for females being 66.54 (range 18-91 years). Gross changes in patients who expired in the first 10 days after disease onset showed a significantly increased mean weight - 1050g, compared to a relatively lower weight in patients expiring more than 10 days after symptom onset - 940g. Histopathology changes were identified as intermittent (developing independent from symptom onset and persisting) - diffuse alveolar damage with hyaline membranes - acute respiratory distress syndrome, endothelitis with vascular degeneration and fibrin thrombi; early (developing within the first week, but persisting) - type II pneumocyte hyperplasia, alveolar cell multinucleation and scant interstitial mononuclear inflammation; intermediate (developing within the late first and second weeks) - Clara cell hyperplasia and late (developing after the second week of symptom onset) - respiratory tract and alveolar squamous cell metaplasia and fibrosis.

Conclusion

COVID-19-associated pulmonary pathology, both gross and histopathology, show a time-related dynamic with persistent early and a myriad of later developing dynamic changes in patients with severe disease. These changes underline both the severity of the condition, as well as the mechanisms and the probability of long-lasting severe complications in patients with post-COVID syndrome.

## Introduction

The novel strain of coronavirus - severe acute respiratory distress syndrome coronavirus 2 (SARS-CoV-2) and the disease it causes clinically (novel coronavirus disease 2019 or COVID-19) present a massive challenge for medical professionals worldwide [1]. The severity of the disease, compared to that of other human coronaviruses, excluding severe acute respiratory distress syndrome coronavirus (SARS/SARS-CoV-1) and middle east respiratory syndrome (MERS/MERS-CoV), have required immense research in the field [2]. Together with the presence of a varying degree of clinical severity, often an unpredictable clinical outcome and lack of specific antiviral treatment, until the introduction of vaccines, have placed medical science in a frenzy towards researching the biological effects of SARS-CoV-2 [3-4].

Despite initial histopathological reports of the pulmonary changes induced by the virus, few published manuscripts have presented an in-depth analysis of the morphology, evolution, and multifaceted features of pulmonary changes [5-7].

## Materials and methods

All patients with real-time reverse transcriptase-polymerase chain reaction (qRT-PCR)-proven COVID-19, subject to an autopsy performed in the period November 2020 - November 2021 were withdrawn from the central pathology repository of the St. Marina University Hospital.

Pulmonary histopathology was compared with the clinical data for patient gender and age, disease onset, symptoms duration, and progression, as well as the presence of concomitant pulmonary diseases.

Morphology of the initial pulmonary section was reevaluated on hematoxylin and eosin, Masson’s trichrome, alcian blue, toluidine blue, periodic acid-Schiff (PAS), phosphotungstic acid hematoxylin (PTAH).

Immunohistochemistry with cluster of differentiation (CD) markers was used to evaluate the type of inflammatory infiltrate with CD3 (polyclonal rabbit, Dako Aligent catalog number IR503; Agilent Technologies, Inc., Santa Clara, California) for T lymphocytes, CD20 (immunoglobulin G monoclonal mouse, Dako Aligent catalog number IR604; Agilent Technologies) for B lymphocytes and CD68 (immunoglobulin G monoclonal mouse, Dako Aligent catalog number IR609; Agilent Technologies) for macrophages. Immunohistochemistry protocols were performed on the automated stainer Autostainer Link 48 (Agilent Technologies) with the EnVision FLEX, High pH (Agilent Technologies) high-sensitivity visualization system using the preprogrammed protocols for the select antibodies. Further immunostains for parenchymal and vascular morphology used were CD34 (immunoglobulin G monoclonal mouse, Dako Aligent catalog number IR632; Agilent Technologies) for endothelial cells, vimentin (immunoglobulin G monoclonal mouse, Dako Aligent catalog number IR630; Agilent Technologies) for fibroblasts and smooth muscle actin (immunoglobulin G monoclonal mouse, Dako Aligent catalog number IR611; Agilent Technologies) for smooth muscle cells.

Morphological and immunohistochemical findings were compared with patient age, gender, and duration of symptoms antemortem, with the goal of establishing the dynamics of histopathological changes within the lungs in patients with severe COVID-19 and their probable long-lasting consequences for survivors.

## Results

A total of 27 autopsy cases fit the established criteria. All cases clinically manifested with severe COVID-19. Autopsies were performed with a mean postmortem interval of 28 hours, range five to 60 hours.

Patient demographics

From the selected 27 cases, n=14 were male and n=13 were female. The mean age in the cohort was 67.44 years (range 18-91 years), with the mean age for males being 68.29 (range 38-80 years) and the mean age for females being 66.54 (range 18-91 years).

Duration of symptoms

The mean duration of symptoms was 16.33 days from their onset to the patients expiring, with a range of two to 35 days. No significant difference was observed in the duration of symptoms between males and females or the different age groups.

Gross pulmonary pathology

Grossly, the lung in all patients presented with a morphology of diffuse alveolar damage (DAD) with hyaline membranes - acute respiratory distress syndrome (ARDS) - parenchymal consolidation, increased weight, and deep blue-reddish color. In patients who expired in the first 10 days after disease onset, the lungs were with a significantly increased mean weight - 1050g (range 780-1400g), compared to relatively lower weight in patients expiring more than 10 days after symptom onset - 940g (range 720-1040g) (Videos 1-2). Furthermore, grossly, the lungs of patients expiring more than 10 days after disease onset had a more grayish-red color to them (Video 2).

**Video 1:** Gross section of the left lung from a patient who expired on day six after symptom onset The lung is enlarged (980g) and bluish-red, with diffuse consolidation

**Video 2:** Gross section of the right lung from a patient who expired on day 15 after symptom onset The lung is enlarged (820g) and grayish-red, with diffuse consolidation

Histopathological changes

Based on the presence of changes and their correspondence to the duration of the symptoms, we were able to separate the changes into four groups - intermittent changes, which can develop at any one time of symptom onset; early changes, which occur with the onset of symptoms and persist for the whole duration of the infection; intermediate changes, which develop after the first week from symptom onset; and late changes, which develop after the second week of disease onset.

**Figure 1:**
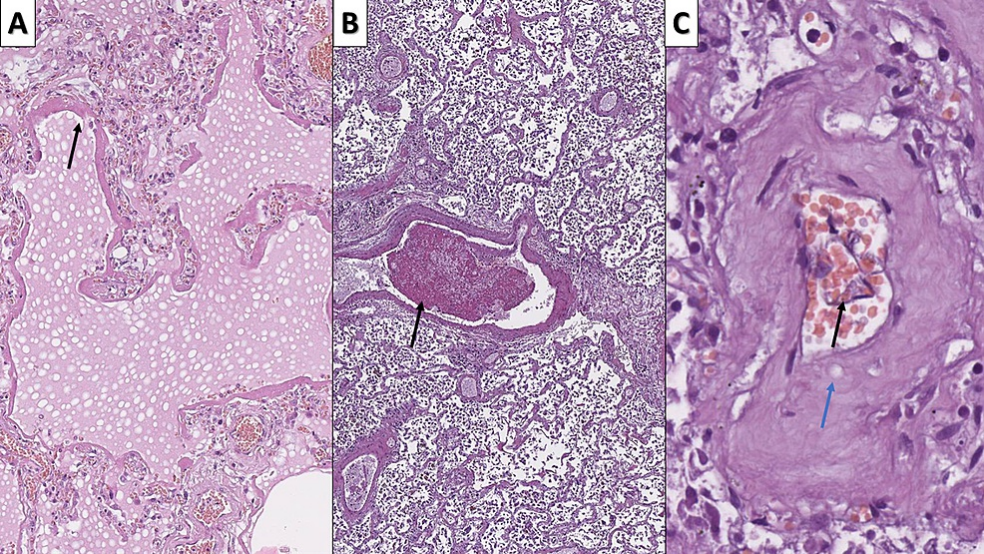
Intermittent changes, associated with COVID-19 A: DAD with pulmonary hyaline membranes (arrow) - ARDS, hematoxylin and eosin, original magnification 100x; B: fibrin thrombi (arrow), hematoxylin and eosin stain, original magnification 40x; C: endothelitis with endothelial delamination into the vascular lumen (arrow) and severe vascular wall edema with subendothelial deposits (blue arrow), hematoxylin and eosin stain, original magnification 400x COVID-19: novel coronavirus disease 2019; DAD: diffuse alveolar damage; ARDS: acute respiratory distress syndrome

Intermittent changes that were observed in the cases, irrespective of the time since symptom onset were DAD (ARDS) (n=20, 74.07%, mean duration of symptoms 13.45 days, range 2-25 days), endothelitis (endothelialitis, intimal arteritis, capillaritis) with vascular wall degeneration (n=27, 100%, mean duration of symptoms 16.33 days, range 2-35 days), and fibrin thrombi (n=20, 74.07%, mean duration of symptoms 16.8 days, range 2-35 days) (Figure 1).

**Figure 2:**
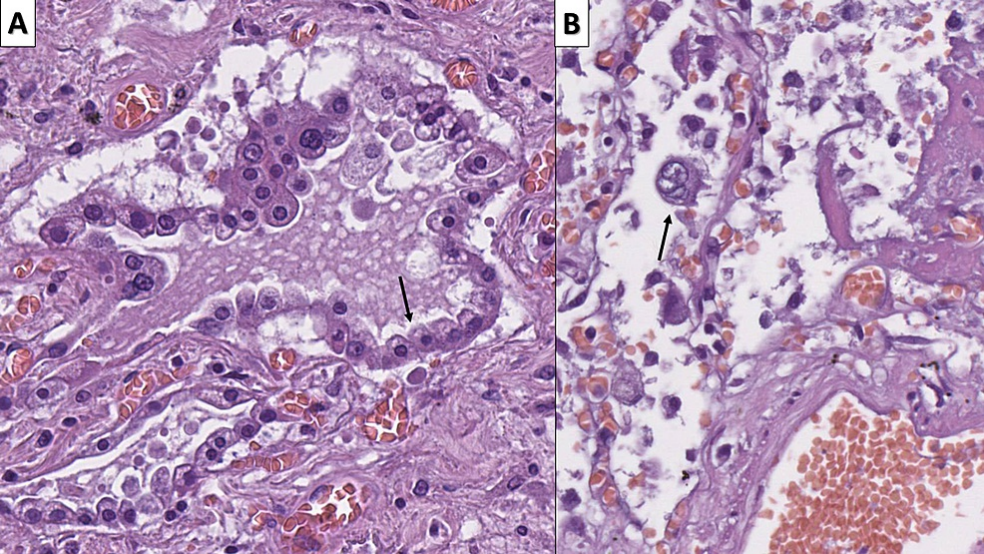
Early COVID-19 associated changes A - type II pneumocyte hyperplasia (arrow), hematoxylin and eosin stain, original magnification 400x; B - multinucleated alveolar cell (arrow), hematoxylin and eosin stain, original magnification 400x COVID-19: novel coronavirus disease 2019

**Figure 3:**
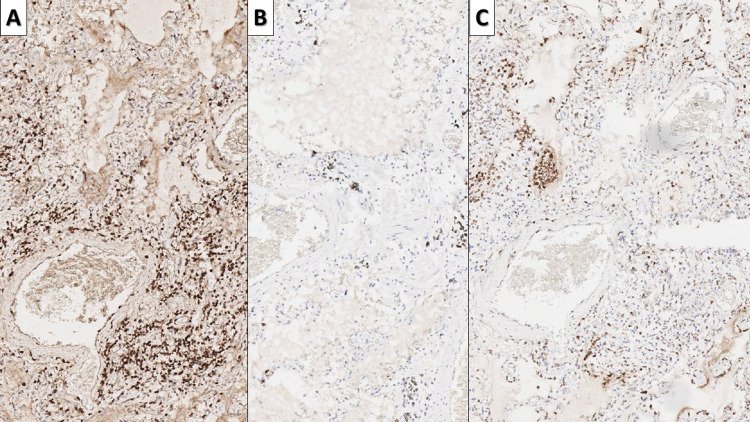
Inflammatory infiltrate in early COVID-19 infection A - interstitial infiltration by T lymphocytes, CD3 immunohistochemistry, original magnification 80x; B - no significant increase in B lymphocytes, CD20 immunohistochemistry, original magnification 80x; C - infiltration by macrophages, CD68 immunohistochemistry, original magnification 80x COVID-19: novel coronavirus disease 2019; CD: cluster of differentiation

The early changes consist of the well-established type two pneumocyte hyperplasia, alveolar cell multinucleation (n=27, 100%, mean duration of symptoms 16.33 days, range 2-35 days), and scant interstitial mononuclear inflammation (Figures 2-3).

The interstitial inflammatory cell was histologically predominantly from lymphocytes and a few macrophages. Immunohistochemistry (IHC) with CD markers showed that the infiltrate predominantly consisted of T lymphocytes (CD3 positive) and macrophages (CD68), with B lymphocytes (CD20 positive) nearly at their physiological levels (Figure 3).

**Figure 4:**
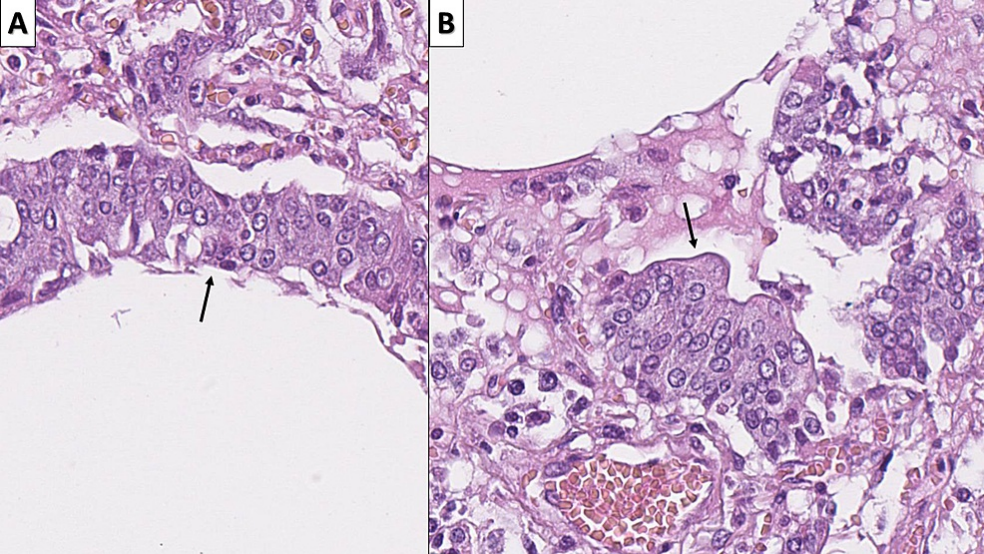
Intermediate changes - Clara cell hyperplasia A: Clara cell hyperplasia (arrow) in respiratory bronchiole, hematoxylin and eosin, original magnification 400x; B: Clara cell hyperplasia (arrow) in alveoli, hematoxylin and eosin stain, original magnification 400x

Intermediate changes comprised predominantly of Clara cell hyperplasia in the terminal respiratory tract and alveoli (n=22, 81.48%, mean duration of symptoms 15.36 days, range 2-25 days) (Figure 4). Clara cells are a type of columnar, non-ciliated respiratory epithelium; however, their hyperplasia seems to replace most of the areas with type II pneumocyte hyperplasia.

**Figure 5:**
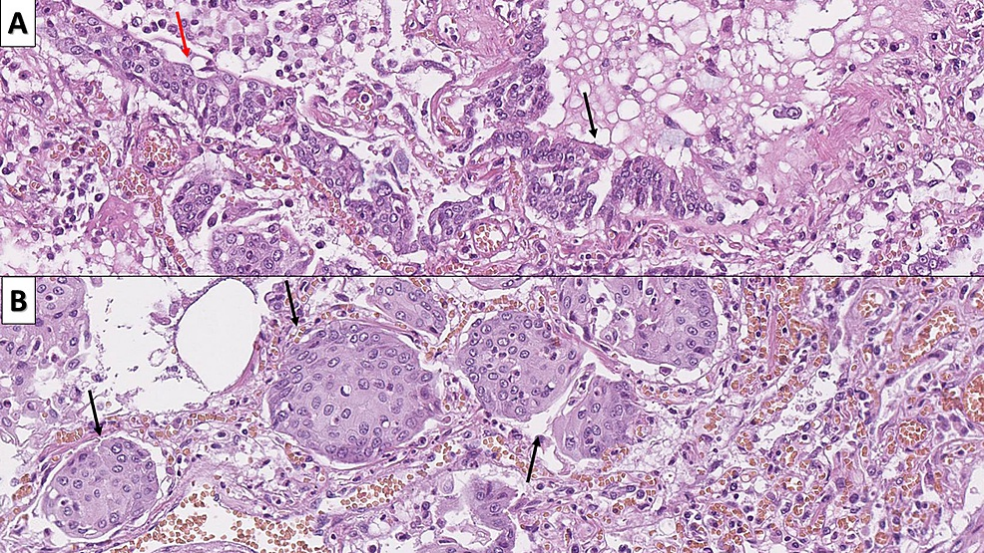
Late changes - squamous cell metaplasia A: transition from Clara cell hyperplasia (arrow) to squamous cell metaplasia (blue arrow), hematoxylin and eosin stain, original magnification 200x; B: intraalveolar squamous cell metaplasia (arrows), hematoxylin and eosin stain, original magnification 400x

**Figure 6:**
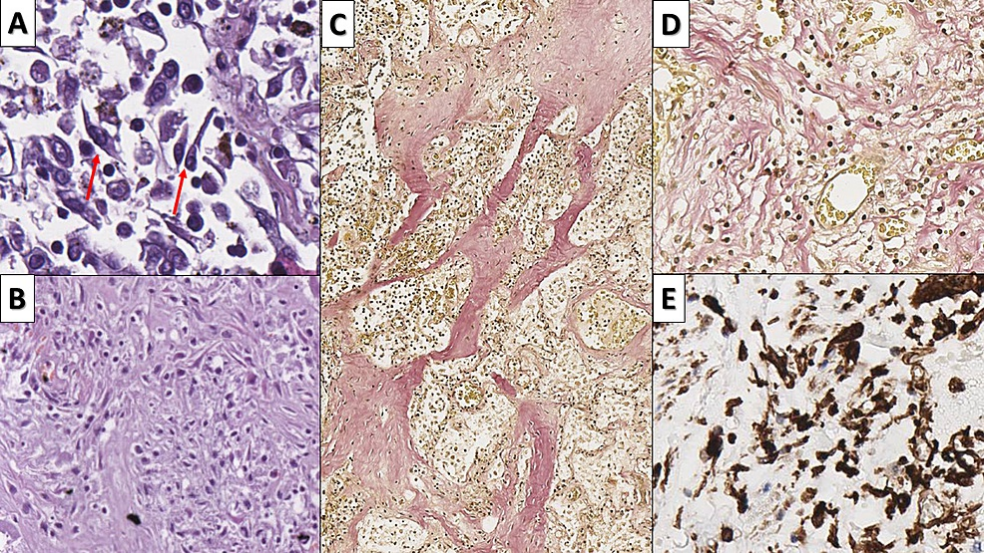
Late changes - fibrosis A: proliferation of intraalveolar fibroblasts (arrows), hematoxylin and eosin stain, original magnification 400x; B: mature fibrous tissue replacing pulmonary parenchyma, hematoxylin and eosin stain, original magnification 200x; C: fibrosis and thickening of alveolar walls (red reaction), Van Gieson stain, original magnification 80x; D: interstitial fibrosis (red reaction), Van Gieson stain, original magnification 200x; E: fibroblast proliferation, Vimentin immunohistochemistry, original magnification 200x

**Figure 7:**
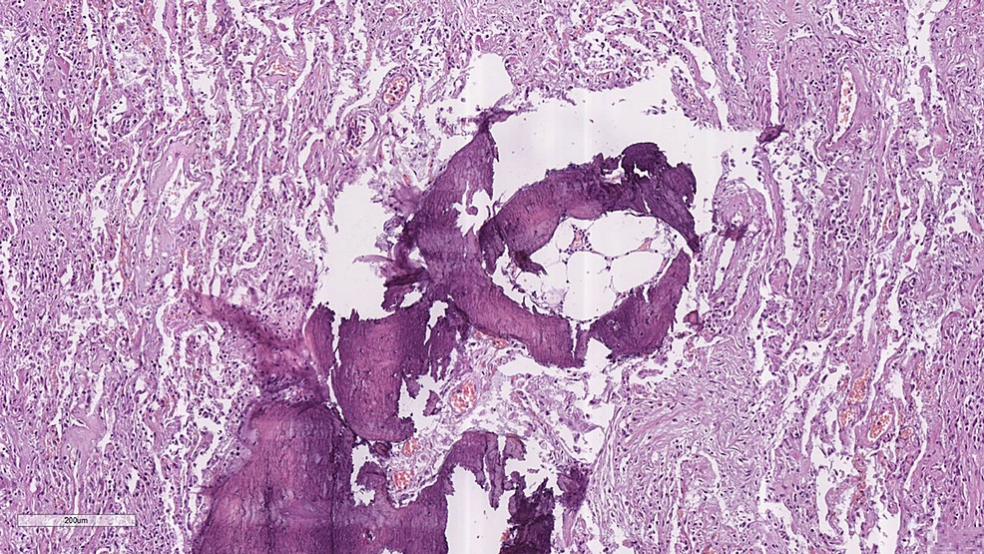
Late changes - fibrosis with osteoid and myeloid metaplasia Proliferating bone matrix, together with bone marrow amidst the pulmonary fibrosis, hematoxylin and eosin stain, original magnification 100x

The most diverse group of changes was that of late changes, developing after the second week from symptom onset (Figures 5-6). The first of these was the transition of Clara cell hyperplasia to squamous cell metaplasia, leading to the formation of intraalveolar squamous cell bulbs (n=10, 37=04%, mean duration of symptoms 18.3 days, range 14-25 days) (Figure 5). Fibrosis (n=20, 74.07%, mean duration of symptoms 18.71 days, range 10-35 days) was observed to develop in several forms, the first one of which with the proliferation of intraalveolar fibroblast and concurrent alveolar obliteration, from the proliferating connective tissue (Figures 6A-6E) and intrinsic fibrosis with thickening of the alveolar walls (Figure 6C). In two cases (n=2, 7.4%), there were foci of osteoid and myeloid metaplasia amidst the fibrosis (Figure 7).

Vascular changes

**Figure 8:**
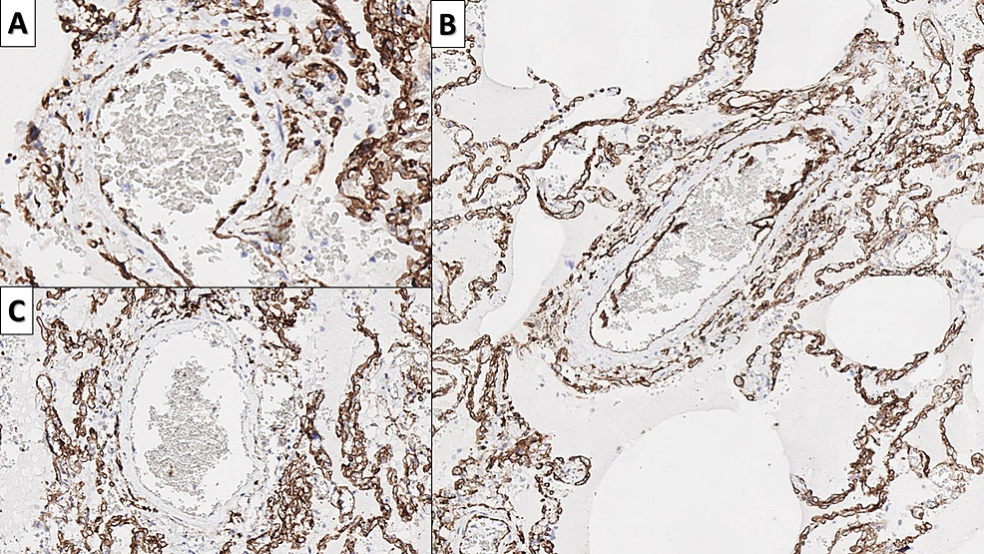
Endothelitis A: endothelial cell bulging, CD34 immunohistochemistry, original magnification 400x; B: endothelial cell delamination, CD34 immunohistochemistry, original magnification 100x; C: complete lack of endothelial cells covering the vascular lumen, CD34 immunohistochemistry, original magnification 200x CD: cluster of differentiation

Of the reported intermittent changes, the endothelitis and vascular wall degeneration were the most multifaceted of all. Endothelitis presented morphologically in several interconnected patterns - endothelial bulging, endothelial delamination, and non-endothelial coated blood vessels, with a concomitant paucity of lymphocytes and macrophages (Figures 1C, 3A-3B, 8]. These forms coexisted not only in the same patient, but also often in neighboring blood vessels, pointing towards a natural continuation and repeat of the process once the endothelium has proliferated to coat the vascular lumen once more. Furthermore, the changes were observed predominantly in large to medium-sized blood vessels, with capillaries being affected predominantly only from endothelial bulging.

**Figure 9:**
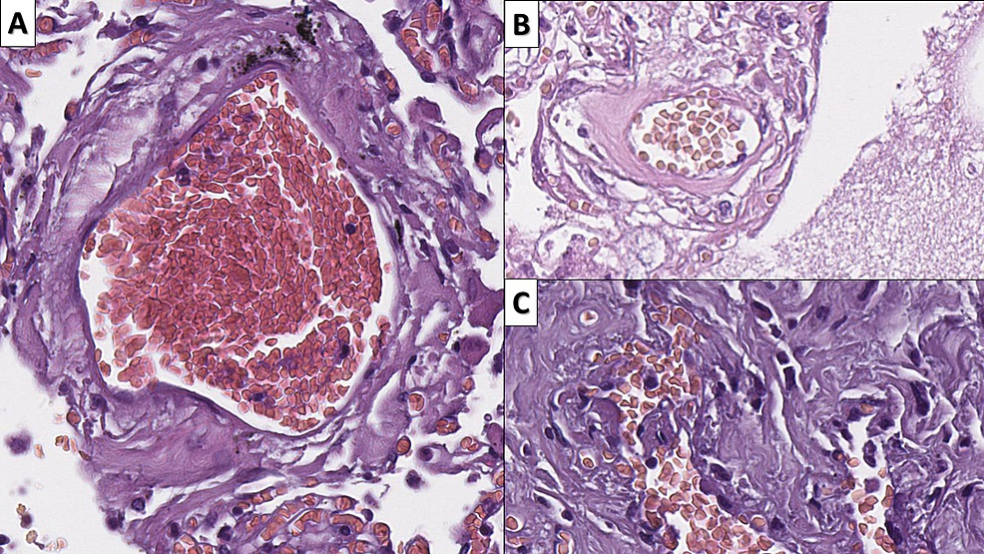
Vascular wall changes A: subendothelial deposits, hematoxylin and eosin stain, original magnification 400x; B: asymmetric vascular wall edema, hematoxylin and eosin stain, original magnification 400x; C: focal fibrinoid necrosis of the vascular wall, hematoxylin and eosin stain, original magnification 400x

**Figure 10:**
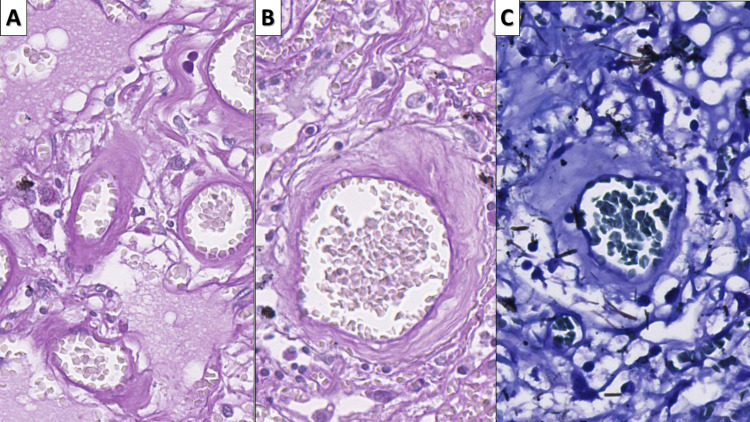
Subendothelial deposits A: protein-rich deposits, PAS stain, original magnification 400x; B: asymmetric edema of the vascular wall, PAS stain, original magnification 400x; C: lack of metachromasia or polysaccharides in asymmetric vascular wall edema, toluidine blue stain, original magnification 400x PAS: periodic acid-Schiff

**Figure 11:**
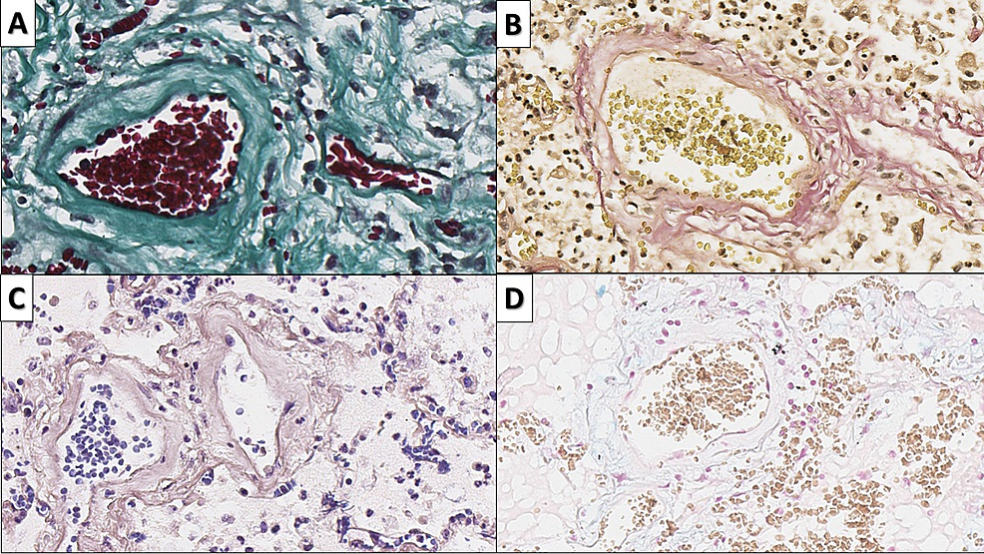
Asymmetric vascular wall edema A: decreased wall density, Masson’s trichrome stain, original magnification 400x; B: disruption of connective tissue structure, Van Gieson stain, original magnification 400x; C: lack of fibrin in asymmetric vascular wall edema, PTAH stain, original magnification 400x; D: lack of mucopolysaccharides is vascular wall edema, Alcian blue stain, original magnification 400x PTAH: phosphotungstic acid hematoxylin

**Figure 12:**
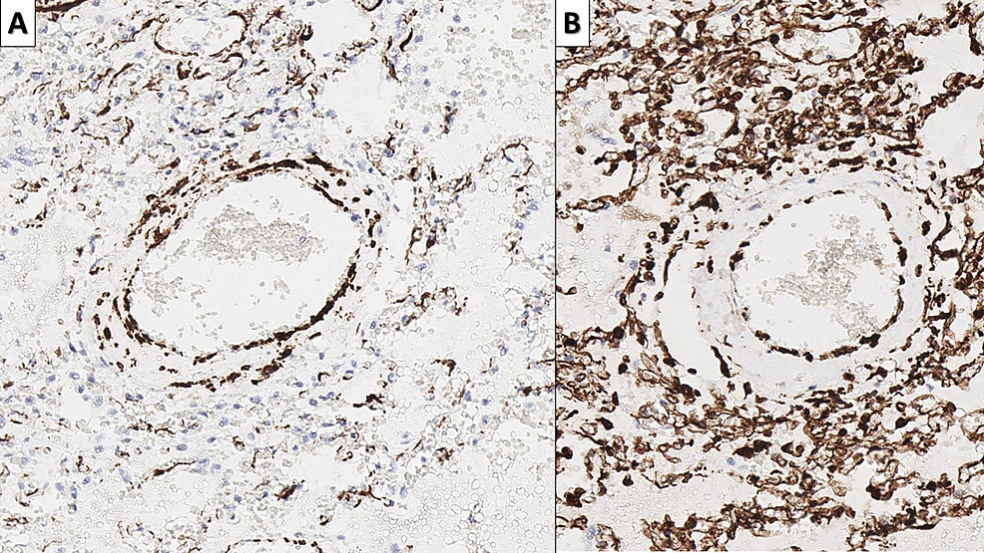
Structural changes in the vascular wall A: distortion of smooth muscle cell arrangement, smooth muscle actin immunohistochemistry, original magnification 200x; B: skip-lesions in subendothelial fibroblast arrangement, Vimentin immunohistochemistry, original magnification 200x

Vascular wall degeneration of medium-sized vessels was also a phenomenon observed in several different patterns in neighboring sections (Figures 9-12). The PAS stain showed protein-rich deposits in some of the blood vessels, akin to hyalinosis, which was also PTAH negative; however, the majority of blood vessels, including those with asymmetric wall thickening, did not show a positive reaction for PAS, PTAH, Masson’s trichrome, Alcian and toluidine blue, confirming the presence of simple focal wall edema (Figures 10-11). Immunohistochemistry for smooth muscle cells with smooth muscle actin and fibroblasts with Vimentin showed a distortion of the smooth muscle architecture of the wall, as well as areas of subendothelial fibroblast arrangement, with the formation of a skip lesion overlying the edematous area of the vascular wall.

Additional findings

Secondary superimposed bacterial infection with purulent inflammation was seen in 74.07% (n=20%, mean duration of symptoms 16.85 days, range 7-35 days) of cases, with other common findings being associated with the already reported changes such as pulmonary infarctions, pulmonary hemorrhages, prominent megacaryocytosis in the pulmonary microcirculation.

Concomitant pulmonary diseases

There were three cases with significant pulmonary comorbidities: an 18-year-old female with pulmonary interstitial involvement from a clinically unrecognized lipid storage disease, a 38-year-old male with clinically unrecognized pulmonary tuberculosis, and a 46-year-old female with pulmonary infiltration from a lymphoproliferative disease and associated pulmonary aspergillosis.

## Discussion

The presented results show a wide set of changes in the morphology of the lung associated with COVID-19, with a relatively specific time-related onset (early, intermediate and late), as well as severe disease-related changes that develop independent of time since disease onset (DAD, thrombosis, and superimposed bacterial infection). Despite our cohort not being representative of the male to female ratio, both in infection and mortality rates, as well as the reported age figures in severe disease and mortality, it underlines the multifaceted morphology of SARS-CoV-2 infection and the myriad of severe acute infection-related complications and the possibility of chronic infection-related pulmonary morbidity [8-9].

The histomorphology of SARS-CoV-2 infection shows viral tropism towards epithelial cells within the pulmonary parenchyma [10-12]. Despite multiple small cohort reports on histopathology being present, most of them focus on the aspect of changes in their alterative, exudative, and proliferative pattern and not on the dynamics of these changes [5,12]. Furthermore, the extent of these changes and the timeframe of their initiation underline not only the myriad of possible life-threatening complications but also the fast evolution of irreversible changes (eg. fibrosis), which are a component of post-COVID syndrome [13].

The most universally observed changes in our cases are type II pneumocyte hyperplasia, alveolar cell multinucleation, and endothelitis [11-12]. Despite them being present in all cases, however, they cannot be considered diagnostic on morphology alone, as type II pneumocyte hyperplasia is observed in multiple infectious and noninfectious pulmonary diseases, ranging from influenza to silicosis, with alveolar multinucleated cell observable in herpes pneumonia and respiratory syncytial virus infections as well [14-17].

Of interest are also the endothelitis and vascular wall changes, which based on their morphology and evolution, can lead to pulmonary vascular hyalinosis, pulmonary hypertension, and chronic cor pulmonale [18]. Despite not focusing on infectious diseases, but on immune-mediated damage in organ transplant rejection, the widest used classification system for endothelitis is the Banff grading system [19]. If applied to the morphological changes observed in the pulmonary vasculature in our cases, the endothelitis, despite the limited loss of the vascular lumen, paucity of subendothelial lymphocytes and intramural macrophages, fits the criteria for grade 3 endothelitis due to the transmural changes in the vascular wall, with loss of smooth muscle cells further suggesting the possibility for severe chronic changes after COVID-19 [19-21]. Furthermore, the presence of these changes predominantly in large to medium-sized blood vessels explains the widely reported thrombotic complications in these patients, with fibrin thrombi, which do not represent embolic disease but rather autonomous pulmonary thrombosis [20].

Of the highest concern regarding the spectrum of histopathological changes are the late changes associated with the infection mainly the diffuse and, in some cases, severe fibrosis as well as the diffuse squamous cell metaplasia in the respiratory bronchioles, alveolar tracts, and alveoli. Fibrosis leads to a reduction of the overall pulmonary vascular volume and right-sided heart overload, which coupled together with the endothelitis of the large vessels can lead to secondary pulmonary hypertension, chronic cor pulmonale, and congestive heart failure and its myriad of perceivable complications as a socially relevant condition [22-23]. It yet remains to be established if the phenomena of fibrosis are related to the inflammation of the pulmonary parenchyma, in the background of limited pulmonary tissue necrosis, a severe decrease of oxygen saturation as a promotion of fibroblast growth, endothelitis related, or a combination of these [24].

Furthermore, it has not yet been established, despite the squamous cell metaplasia being reported by other authors as well, whether the squamous cell metaplasia as an observable phenomenon in acute infection is a reversible process. Despite following the typical pattern of inflammatory induces squamous cell metaplasia, which develops in the background of Clara cell hyperplasia, replacing both the standard ciliated respiratory epithelium and the pneumocytes within the alveoli, this process develops rapidly and in atypical places, as well as being diffuse [25]. Whilst squamous cell metaplasia is a relatively common finding in the elderly, especially those with chronic pulmonary inflammatory-mediated damage, it typically affects single foci within the large to medium-sized bronchi, whilst here the process is much more diffuse and affects much smaller structures within the respiratory system. As the squamous cell metaplasia itself is a premalignant condition, this places the phenomenon under high morphological concern for the development of squamous cell carcinoma, if the changes are irreversible or take a long time to disappear [25-26]. Furthermore, these can be a diagnostic challenge as well, due to their relatively atypical location, which can lead to pulmonary biopsy overinterpretation and the diagnosis of squamous cell carcinoma in such patients.

Study limitations

As already mentioned, the reported gender and age of our cohort are not representative of morbidity and mortality rates in COVID-19. Furthermore, as with our study and multiple other studies, the cohort size is small compared to the overall mortality figures. This necessitates future large-scale studies on disease dynamics and the morphological aspects associated with it. Furthermore, a standardized specimen obtaining procedure should also be implemented, which will limit postmortem time to the autopsy, allow for proper fixation of multiple tissue fragments obtained from specific parts of the pulmonary parenchyma with the goal of better understanding the topography of the process. As universal testing rates are increasing, this would also allow future studies to report on changes associated with time from disease onset, unlike our study, which used patient-reported symptom onset, which is a significantly shorter period.

Future directions

It is also important to note that the depicted changes are present in patients with severe disease, and it remains to be seen if they are also present in cases with moderate and mild disease course. Furthermore, it is vital that patients with post-COVID syndrome, subject to autopsy or biopsy, be interpreted in depth, with the goal of establishing the dynamics of these changes and their progression or regression, with regards to further perceivable complications.

## Conclusions

SARS-CoV-2 infection and the disease it causes, COVID-19, cause severe, if non-specific for the condition, morphological changes in the pulmonary parenchyma, resulting in a well-established spectrum of changes. In our study, we focused on the dynamics of these changes - early, intermediate, late, and intermittent. These changes and their onset correlate in time to the onset of clinically established complications in cases of severe disease but can also shed light on the long-lasting complications of the disease in survivors. Taken together, they implicate the need for infection prophylaxis and long-time monitoring of survivors with severe disease, with the goal of early detection of the development of severe post-COVID syndrome complications.
